# Alternative cancer cures

**DOI:** 10.1054/bjoc.2001.1989

**Published:** 2001-09

**Authors:** E Ernst

**Affiliations:** Department of Complementary Medicine, School of Postgraduate Medicine and Health Sciences, University of Exeter, 25 Victoria Park Road, Exeter, EX2 4NT


BJOC 01-1989 781-782 20/8/01 3:51 pm Page 781

*British Journal of Cancer* (2001) **85**(5), 781–782
© 2001 Cancer Research Campaign
doi: 10.1054/ bjoc.2001.1989, available online at http://www.idealibrary.com on IDEAL
http://www.bjcancer.com

Letters to the Editor

### Alternative cancer cures

**Sir**,

Cancer patients make ample use of complementary and alternative medicine (CAM), often being led to hope for a miracle cure (Ernst and Cassileth, 1998). The advent of the internet renders patients all the more vulnerable. Serious harm and even fatalities of cancer patients following instructions for using CAM from the internet are reported with depressing regularity (e.g. (Hainer et al, 2000)). This short article is aimed at enhancing oncologists' understanding this complex area.

Alternative cancer cures (ACCs) typically have a common life cycle (Ernst, 2000). At the origin of almost every ACC is a charismatic individual who claims to have found the answer to cancer. He (the male sex seems to dominate) often supports his claims with pseudoscientific evidence referring to (but rarely presenting) many cured patients. Thus he soon gathers ardent supporters who lobby for a wider acceptance of this ACC. The pressure on the medical establishment increases to a point where the treatment is finally submitted to adequate testing. When the results turn out to be negative, the ACC's proponents argue that the investigations were not done properly. In fact, they were set up to generate a negative result so that the commercial interests of orthodoxy would not be threatened. A conspiracy theory is thus born, and the ACC lives on in the 'alternative underground'. Henceforward the gap between the two camps is destined to widen; mainstream oncologists would claim that they have done their duty by submitting the ACC to rigorous tests which have demonstrated it to be useless. Supporters of the ACC dwell on their theory of a conspiracy against them and continue to state that an effective cure is being shamelessly suppressed. Both parties see themselves as winners, but sadly, the loser often turns out to be the patient!

Receiving a diagnosis of cancer is surely one of the most shattering experiences one can possibly imagine. As soon as the initial psychoemotional trauma is over, many patients want to assess their options. This is when they access the world of ACCs, where the most fantastic claims supported by seemingly compelling anecdotes and pseudoscience abound. It is easy to see how many patients become deeply confused. Who can they turn to? Unfortunately, help is hard to find. Oncologists are usually not well informed about CAM and often dismiss the entire field outright (Newell and Sanson-Fisher, 2000). This attitude can confirm the above-mentioned conspiracy theory in the eyes of the patient. CAM books are as plentiful as they are unreliable (Ernst and Armstrong, 1998). Health food stores readily provide advice but this information is primarily motivated by the desire to increase sales and can put cancer patients at considerable risk (Cook Gotay and Dumitriu, 2000). CAM providers are sympathetic but can they be trusted?

Patients thus face lonely and difficult decisions. Many opt to follow the alternative route and invest in expensive, demanding, ineffective and hazardous ACCs (e.g. (Hainer et al, 2000; Richardson et al, 2000; von Gruenigen and Hopkins, 2000)) sometimes abandoning orthodox treatments altogether (e.g. (Hainer et al, 2000)). The decision to try CAM might be a marker either for the psychological distress these patients feel (Burstein et al, 1999) or for their active coping behaviour (Sollner et al, 2000). Of particular concern is the fact that, in about 50% of the cases, patients do not inform their doctor about the decision to try CAM (e.g. (Begbie et al, 1996; Boon et al, 2000)).

Oncologists may react with surprise, anger, sadness or apathy. They feel sure to know the truth: no single treatment is likely to cure all cancers; the plausibility of these ACCs is close to zero; the evidence is clearly against them (Ernst et al, 2001). Most importantly, they know that orthodox medicine is far from being a conspiratory society; it would not hesitate in adopting a cancer cure that is demonstrably effective, or test a treatment that holds any reasonable promise at all. Thus, in the view of mainstream oncologists, ACCs represent a contradiction in terms. The trouble is, however, that many desperate cancer patients are not being convinced. Perhaps they don't want to be convinced, but may be mainstream medicine is also bad at getting its points across?

A lesson seems to emerge: doctors should ask their patients about CAM-use, be informed about CAM, and openly pass on their knowledge to their cancer patients, ensuring that they are understood (Eisenberg, 1997). To achieve this we need empathy, sympathy and time. Rather than damning all CAM outright, it might be helpful to point out that some forms of CAM can be useful not for curing cancer but for supportive and palliative cancer care (Ernst, 2001). Insistence on taking the moral high ground of 'good science' is probably counterproductive and may constitute the very impetus that drives many patients towards CAM. In that sense, every cancer patient harmed through an ACC also represents a failure of orthodox medicine.

**E Ernst**
Department of Complementary Medicine, School of Postgraduate Medicine and Health Sciences, University of Exeter, 25 Victoria Park Road, Exeter EX2 4NT

### REFERENCES

Begbie SD, Kerestes ZL and Bell DR (1996) Patterns of alternative medicine use by cancer patients. *Med J Australia* **165**: 545–548
Boon H, Stewart M, Kennard M et al (2000) Use of complementary/alternative medicine by breast cancer survivors i Ontario: prevalence and perceptions. *J Clin Oncol* **18**: 2515–2521
Burstein HJ, Gelber S, Guadagnoli E and Weeks JC (1999) Use of alternative medicine by women with early-stage breast cancer. *New Engl J Med* **340**: 1733–1739
Cook Gotay C and Dumitriu D (2000) Health food store recommendations for breast cancer patients. *Arch Fam Med* **9**: 692–698
Eisenberg DM (1997) Advising patients who seek alternative medical therapies. *Ann Intern Med* **127**: 61–69
Ernst E (2000) The role of complementary and alternative medicine in cancer. *Lancet Oncology* **1**: 176–180
Ernst E (2001) A primer of complementary and alternative medicine commonly used by cancer patients. *Med J Aust* **174**: 88–92
Ernst E and Armstrong NC (1998) Lay books on complementary/alternative medicine: a risk factor for good health. *Int J Risk Safety Med* **11**: 209–215
Ernst E and Cassileth BR (1998) The prevalence of complementary/alternative medicine in cancer: A systematic review. *Cancer* **83**: 777–782
Ernst E, Eisenberg D, Pittler MH, Stevinson C and White AR (2001) The desktop guide to complementary and alternative medicine. Edinburgh.

BJOC 01-1989 781-782  20/8/01  3:51 pm  Page 782

Hainer MI, Naoky T, Komura ST and Chiu C (2000) Fatal hepatorenal failure associated with hydrazine sulfate. *Ann Intern Med* **133**: 877–880

Newell S and Sanson-Fisher RW (2000) Australian oncologists' self-reported knowledge and attitudes about non-traditional therapies used by cancer patients. *MJA* **172**: 110–113

Richardson MA, Sanders MPH, Tamayo C, Perez C and Palmer JL (2000) Flor-Essence® herbal tonic use in North America: a profile of general consumers and cancer patients. *Herbalgram* **50**: 40–46

Sollner W, Maislinger S, DeVries A, Steixner E, Rumpold G and Lukas P (2000) Use of complementary and alternative medicine by cancer patients is not associated with perceived distress or poor compliance with standard treatment but with active coping behavior. *Cancer* **89**: 873–880

von Gruenigen V and Hopkins MP (2000) Alternative medicine in gynecologic oncology: a case report. *Gynecol Oncol* **77**: 190–192

*© 2001 Cancer Research Campaign*

